# Quercetin and 5-Fu Loaded Chitosan Nanoparticles Trigger Cell-Cycle Arrest and Induce Apoptosis in HCT116 Cells via Modulation of the p53/p21 Axis

**DOI:** 10.1021/acsomega.3c03933

**Published:** 2023-09-28

**Authors:** Sanjib Das, Moumita Saha, Lokesh Chandra Mahata, Arya China, Niloy Chatterjee, Krishna Das Saha

**Affiliations:** † Cancer Biology and Inflammatory Disorder Division, CSIR- Indian Institute of Chemical Biology, Jadavpur, Kolkata 700032, West Bengal, India; ‡ Department of Pharmaceutical Technology, Maulana Abul Kalam Azad University of Technology, Haringhata, Nadia 741249, West Bengal, India; § Laboratory of Food Science and Technology, Food and Nutrition, University of Calcutta, 20B, Judges Court Road, Kolkata 700027, West Bengal, India; ∥ Centre for Research in Nanoscience & Nanotechnology, University of Calcutta, JD-2, Sector-III, Salt Lake City, Kolkata 700098, West Bengal, India

## Abstract

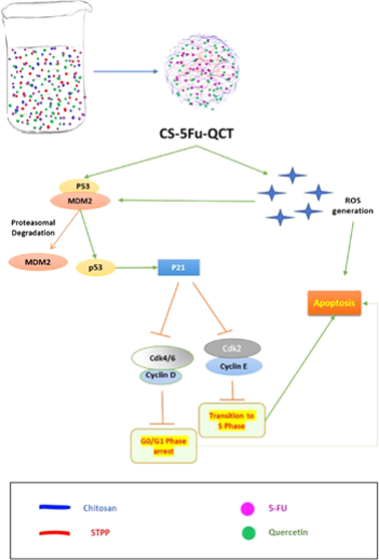

Nanoparticles (NPs) are encapsulating agents that exist in the nanometer range. They can be classified into different classes based on their properties, shapes, or sizes. Metal NPs, fullerenes, polymeric NPs, ceramic NPs, and luminescent nanoporous hybrid materials are only a few examples. This study explored the anticancer potential of quercetin and 5-fluorouracil-encapsulated chitosan nanoparticles (CS-5-FU-QCT NPs). The nanoparticles were prepared by ionic gelation, and their efficacy and mechanism of action were examined. CS-5-FU-QCT NPs were characterized using dynamic light scattering (DLS), atomic force microscopy (AFM), UV–visible spectroscopy, and Fourier transform infrared spectroscopy (FTIR); cytotoxicity was analyzed using an MTT assay. Cells were treated with CS-5-FU-QCT NPs and incubated for 12, 24, and 36 h, and apoptosis analysis (using Annexin V/FITC), cell-cycle analysis, Western blotting, and confocal microscopic analysis were performed. Biophysical analysis revealed that the CS-5-FU-QCT NPs fall in the range of 300–400 nm with a near-spherical shape. The i*n vitro* drug release profile indicates sustained release of drugs over a period of about 36 h. The cytotoxicity of CS-5-FU-QCT NPs was more prominent in HCT116 cells than in other cancer cells. This particular nanoformulation caused G0/G1 phase cell-cycle arrest in HCT116 cells and induced intracellular ROS generation, thereby causing apoptosis. It also downregulated Bcl2, cyclin D1, and Cdk4 and upregulated BAX, p53, and p21, causing cell-cycle arrest and apoptosis. In summary, CS-5-FU-QCT NPs hindered proliferation of HCT116 cells via ROS generation and altered the expression of key proteins in the p53/p21 axis and apoptotic machinery in a time-dependent manner.

## Introduction

Nanoparticles (NPs) are nanometer-sized particles that act as encapsulating agents. They can be classified according to their properties, shapes, or sizes. Examples include metal NPs, fullerenes, polymeric NPs, ceramic NPs, and luminescent nanoporous hybrid materials..^1,2^ Nanomedicine is concerned with nanodelivery systems that act as a controlled carrier of therapeutic agents. NPs have recently been used in the treatment of various diseases as chemotherapeutic agents, immunotherapeutic agents, biological agents, and others.^3^ Cancer represents uncontrolled cell growth, leading to tumor formation by alteration of intracellular signaling pathways and manipulating apoptosis. Different classes of cancer are defined by their originating cell types, for example, epithelial cell cancer as carcinoma, connective tissue cancer as sarcoma, cancer from the hematopoietic cells as lymphoma and leukemia, and embryonic tissue cancer as blastoma.^4^ The World Health Organization has reported that 10 million people have died due to cancer in 2020.^5^ However, among the different types of cancers, colon and rectum cancer (916,000 deaths) constitute the second most common cause of death after lung cancer (1.80 million deaths). Liver, stomach, and breast cancer are the other leading causes of death. In 2023, the United States is expected to have 1,958,310 new cancer cases and 609,820 cancer deaths.^6^ One of the challenges in cancer therapy is poor therapeutic targeting, which results in severe adverse effects on normal tissues. To address this, nanoscale drug delivery systems have provided an alternative route for improving the therapeutic potential of various drugs and bioactive compounds via the enhanced permeability and retention effect.^7^ Chitosan is a chitin derivative derived from crustacean shells. Removal of the acetate moiety from chitin produces chitosan. Chitosan, a biodegradable and biocompatible polymer, has been used as a carrier in polymeric nanoparticles for drug delivery via various routes. Owing to its less toxicity in both *in vitro* and *in vivo* models, chitosan-coated nanoparticles are used in a wide array of drug delivery applications.^8^ 5-Fu is a short-half-life antimetabolite chemotherapeutic drug approved by the US Food and Drug Administration (FDA) to treat various types of cancers. It is a pyrimidine analogue having a heterocyclic aromatic nature. When used as a single chemotherapy drug, its mechanism of action is based on metabolite incorporation into RNA, which inhibits RNA replication and blocks thymidylate synthase (TS), which hinders tumor cell growth by blocking DNA replication.^9^ It has been widely used for decades in the treatment of different cancers, including colon and breast cancers. However, it has limited clinical applications due to drug resistance and a lower response rate.^4^ At present, 5-Fu is employed in conjunction with other chemotherapeutic drugs, which improves the cytotoxic effect against tumors due to a synergistic effect.^10^ Therefore, encapsulating it in conjugation with other chemotherapeutic agents within nanomaterials can be a promising approach to enhance its effectiveness by increasing the target-specific drug delivery. 3,30,40,5,7-Pentahydroxylflavone or quercetin (QCT) is a secondary metabolite present in the plant kingdom. QCT has antioxidant properties due to the presence of phenolic hydroxyl groups at the B-ring and the 3-position. Its anticarcinogenic properties have been attributed to its antioxidant effect, modification of intracellular signal transduction, and inhibition of carcinogen-activating enzymes.^11−13^ Jeong et al. focused on the physiologically viable dose of quercetin on the cell-specific inhibition of cancer proliferation by quercetin through arrest of the cell cycle at the G1/S phase by trapping E2F1.^14^ Studies also reported the ability of QCT to inhibit lipid peroxidation.^15,16^ QCT-mediated induction of activation of p38/MAPK has also been reported.^16^ QCT-based nanoformulations have shown improved bioavailability, solubility, biodistribution, and target specificity.^17^ Roy et al., 2021, developed functional chitosan-/quercetin-based nanocomposite films, which showed potent antioxidant activity.^18^ These reports together promote QCT-based nanocompounds as potent chemotherapeutic agents with diverse anticancer properties. Various studies have portrayed QCT and 5-Fu as pro-oxidants, which induce intracellular ROS generation.^19,20^ In general, cancer cells frequently release cytotoxic reactive oxygen species (ROS) in response to chemotherapeutic drugs.^21^ ROS is an important regulator of DNA damage.^22^ Superoxide anion radicals, hydrogen peroxide, singlet oxygen, and the highly reactive hydroxyl radical are all examples of ROS. The harmful effects of oxygen are thought to be caused by its metabolic reduction to these highly reactive and toxic species.^23^ In 2016, Redza-Dutordoir et al. reported that increased cellular ROS levels can damage proteins, nucleic acids, lipids, membranes, and organelles, leading to the activation of cell death processes like apoptosis.^24^ Apoptosis is a type of programmed cell death distinguished by distinct morphological characteristics and energy-dependent mechanisms.^25^ Reports from previous studies suggest that QCT can activate apoptosis via mitochondrial pathways, thereby regressing tumor size.^26^ Studies also reported that apoptosis is induced by 5-Fu in colorectal cancer cells through the activation of caspase-9 and PKCδ, where the levels of PKCδ activation may determine 5-Fu sensitivity of the cells.^27^ P21 and p27 are two important proteins that inhibit cell-cycle progression through inhibition of G1 or G1/S phase cyclin Cdk complexes like Cyclin D-Cdk4/6 or Cyclin E-Cdk2.^28^ Quercetin has been reported to inhibit cyclin D and increase the expression of p21, p27, and p53 to induce G1 growth arrest.^29^ 5-Fu acts by specifically inhibiting S phase cells.^30^ Choi et al. reported that 5-Fu administration on DLD-1 cells for 72 h resulted in an accumulation of cells in the S phase. Administration of chloroquine prior to 5-Fu induced more G1 arrest than 5-Fu alone. Furthermore, the combined effect of 5-Fu and chloroquine resulted in the decreased expression of CDK2 and cyclin D1 and the increased expression of p27 and p53, with small changes in cyclin A, Cdk4, and cyclin E protein levels, suggesting a 5-Fu-induced disruption of the cell cycle.^31^

Combination therapy can overcome cross-resistance and achieve synergistic effects to enhance therapeutic efficacy while minimizing toxicities. The synergistic effect of two (or more) compounds targeting different diseases, genes, or cell-cycle checkpoints in cancer progression is advantageous in combination therapy to increase the chances of cancer elimination. The delivery of numerous therapeutic agents with different physicochemical and therapeutic properties in single NPs is referred to as NP-mediated combination therapy.^32^ Li et al. developed chitosan nanoparticles to codeliver the chemotherapeutic agents 5-fluorouracil and leucovorin in the treatment of colon cancer.^33^ Similarly, Chaitra et al. loaded quercetin and doxorubicin in chitosan-based nanocarriers.^34^ Because of the anticarcinogenic and pro-oxidant properties of 5-Fu and QCT, we hypothesize that when coencapsulated in chitosan, they can be an effective partner in combatting carcinogenicity by inducing specific signaling pathways in cancer cells. In this context, CS-5Fu-QCT nanoparticles were created, their physicochemical properties were determined, and their biological efficacy against cancer was determined.

## Materials and Methods

### Chemicals

Chitosan, sodium tripolyphosphate (STPP) (Cat. No. 238503, Sigma-Aldrich), 5-fluorouracil (5-Fu) (Cat. No. F6627, Merck, Germany), quercetin (QCT) (Cat. No. Q4951 Sigma-Aldrich), 3-(4,5-dimethylthiazol-2-yl)-2,5-diphenyltetrazolium bromide (MTT) (Cat. No. M6494, Thermofischer Scientific), dimethyl sulfoxide (DMSO) (Cat. No. 34869 Merck Lifescience, Germany), propidium iodide (PI) (Cat. No. P3566, ThermoFischer Sci.), H_2_DCF-DA (Cat. No. D6883, Merck, Germany), ethanol, phosphate-buffered saline (PBS), and double distilled water were used. HepG2, HCT116, HeLa, and Hek293 cells were obtained from the National Centre for Cell Science (NCCS), Pune, Govt. of India. Fetal bovine serum (FBS) (Cat. No. 26140087, Gibco), Dulbecco’s modified Eagle’s medium (DMEM) (Cat. No. 12491015, Thermofischer Scientific), Pen-strep (penicillin and streptomycin) (Cat. No. 15140122, Gibco), and trypsin (Cat. No. 25300054, Gibco) were also used.

### Methods

#### Cell Culture

HepG2, HCT116, HeLa, and Hek293 cell lines were cultured in DMEM (Dulbecco’s modified Eagle’s medium) supplemented with 10% fetal bovine serum (Cat. No. 26140087, Gibco, Thermofischer sci.) and 1% Pen-strep under 37 °C and 5% CO_2_.

#### Preparation of CS-5Fu-QCT NPs

5-Fu and QCT-loaded CS NPs were synthesized using an ionic gelation method. Briefly, an aqueous solution of 1% (v/v) glacial acetic acid was used to dissolve chitosan (CS) at 2 mg/mL concentration and stirred to obtain a clear solution. To obtain the highest encapsulation efficiency, 5-Fu and QCT were used in a 2:1 ratio, respectively, according to the methods of David et al. with some modifications.^4^ Different concentrations of 5-Fu and quercetin were prepared and added drop-by-drop using a microsyringe to the CS solution with continuous stirring. To this solution, 2 mg/mL STTP solution was added dropwise as a cross-linking agent and continuously stirred at room temperature overnight until the formation of an opalescent suspension. Then, the suspension was ultracentrifuged at 20,000 rpm for 20 min to discard the unconjugated QCT and 5-Fu. The pellet was collected and lyophilized to obtain the nanoparticles in the dried form.

#### Characterization of NPs

##### Analysis of Particle Size Using Dynamic Light Scattering (DLS)

The average mean particle size of the prepared CS-5FU-QCT nanoparticles was evaluated by the dynamic light scattering technique using a Zetasizer Nano ZS (Malvern Instruments, Malvern, U.K.). Lyophilized CS-5Fu-QCT NPs were suspended in doubled distilled water and filtered through a Millex-GV Syringe Filter 0.22 μm (Cat. No. SLGV033RS, Merck, Millipore, Germany); 600 μL of the filtrate was taken into the cuvette for analysis.

##### Atomic Force Microscopic (AFM) Analysis of CS-5FU-QCT NPs

The surface morphology and particle diameter of freshly prepared nanoparticles were analyzed using AFM (Agilent Technologies). Freshly prepared nanoparticles were diluted and suspended in distilled water and then filtered using a 0.22 μm membrane filter; 10 μL of the dissolved NPs was placed in a cleaved mica sheet and observed using AFM, after which images were analyzed.

##### Fourier Transform Infrared (FTIR) Spectroscopy Analysis

To determine the functional groups present in the CS, QCT, 5-FU, and prepared QCT-5FU loaded nanoparticles were identified using an FTIR spectrometer (Bruker) within the range of 400–4000 cm^–1^. The lyophilized nanoparticles were placed in an IR probe for analysis.

##### Analysis of Particle Morphology Using Transmission Electron Microscopy

The morphology and nanostructure of CS-5FU-QCT NPs were examined by a transmission electron microscope (TEM, JEM-2100 Plus, JEOL Ltd., Tokyo, Japan). The nanoparticle samples were diluted in a 1:100 ratio using Milli-Q water before adding 10 μL of the diluted sample over a copper grid (300-mesh size) (GS Cu 300C, ProSciTech, Qld, Australia) followed by air-drying at ambient temperature. Then, 1% (w/v) phosphotungstic acid (SRL Pvt. Ltd., Mumbai, India) was used as a negative staining agent to stain the grid for 1 min. The stained grids were completely dried in a vacuum before being placed onto the cryo-holder in the TEM instrument.

##### Encapsulation Efficiency Analysis

To determine the encapsulation efficiency, a freshly prepared CS-5FU-QCT NP solution was taken and ultracentrifuged at 20,000 rpm for 20 min. The supernatant was collected, and the absorbance was measured using UV–visible spectroscopy (V-730 UV–visible spectrophotometer, JASCO Inc., Japan) at 265 nm for 5-Fu and at 380 nm for QCT. From the absorbance values, the quantity of drugs was determined using a standard calibration curve of 5-Fu and QCT. The encapsulation efficiency was then calculated using the following formula
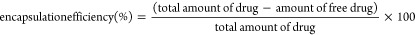


Drug loading of the prepared nanoparticles was calculated using the following formula^35^
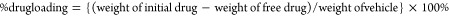


##### Drug Release Study

After activation of the dialysis tubing, freshly prepared drug-loaded NPs were placed in the dialysis tube, and both ends of the tube were sealed using dialysis tubing closure. This was followed by placing the tube in PBS (pH 7.4) at temperature 37 °C. Samples were withdrawn from the solution at specific intervals, and the same amount of fresh PBS was added. Using a UV–vis spectrophotometer (V-730 UV–visible spectrophotometer, JASCO Inc., Japan), absorbance was taken at 265 nm for 5-Fu and at 380 nm for QCT. From the standard curve of 5-Fu and QCT, the concentration of each was determined.

##### Cell Viability Assay

To determine the cell viability using 3-(4,5-dimethylthiazol-2-yl)-2,5-diphenyltetrazolium bromide (MTT) dye, HepG2, HeLa, HCT116, and Hek293 cells were seeded in 96-wale plates and treated with different concentrations with CS-5Fu-QCT NPs. This was followed by incubation of the plate for 24 h. 30 μL of MTT solution was added to each well and then incubated at 37 °C for 4 h. To solubilize the generated purple formazan, DMSO was added. The absorbance was then analyzed using an ELISA reader (Model 550, Ultramark, BioRad) at a wavelength of 595 nm, and the IC_50_ value was calculated.

##### Analysis of Apoptosis via Annexin v-FITC Using Flow cytometry

Cells were seeded for 24 h, which were then treated with CS-5Fu-QCT NPs. Cells were then subjected to dual staining using recombinant FITC-conjugated Annexin V and PI by an Annexin V/FITC Apoptosis Detection kit (Cat. No. V13242, Invitrogen) at different time intervals followed by flow cytometry (BD FACS calibur, BD Biosciences).

##### Confocal Microscopy

The cells were seeded in a 6-well plate and treated with CS-5Fu-QCT NPs followed by incubation for 12, 24, and 36 h. Cells were then washed two times using 1× PBS for 10 min. Then, the cells were rinsed using a blocking buffer solution. Cells were then incubated overnight at 4 °C with primary antibodies against p53 (Cat. No. sc-47698, SCBT), p21 (Cat. No. sc-6246, SCBT), Bcl2 (Cat. No. sc-7382, SCBT), and BAX (Cat. No. sc-7480, SCBT). Then, the cells were washed and incubated with the fluorophore conjugated secondary antibodies AF 647 (Cat. No. sc-516609, SCBT, Cat. No. 4414, Cell Signal Technology; Cat. No. A-21447, ThermoFischer Sci.), CFL555 (Cat. No. sc-516242, sc-362267, sc-516249, SCBT), and FITC (Cat. No. sc-2010, SCBT). After that, the slides were taken and counterstained using DAPI. Then, the coverslips were mounted on slides. Stained cells were observed under a confocal laser scanning microscope (Zeiss).

##### Measurement of Intracellular ROS Generation by H_2_DCF-DA

Intracellular ROS generation was determined by H_2_DCF-DA. Intracellular ROS oxidizes H2DCF-DA to 2,7-dichloro-fluorescein (DCF), which is a fluorescent compound. First, HCT116 cells were treated with drug-loaded chitosan nanoparticles and incubated for 12, 24, and 36 h. Cells were pelleted down and resuspended in 400 μL of PBS. Then, 10 mM H_2_DCF-DA solution was added and kept for 15 min at room temperature. Samples were collected in an FACS tube and analyzed using flow cytometry (BD Bioscience).

##### Analysis of Cell Cycle

Analysis of DNA content is among the versatile approaches used to detect cell-cycle phases.^36^ In this study, we also employed the same detection procedure. The principle is that the DNA content of G1 and G0 cells is half that of G2 and M cells. Briefly, the DNA content of cells is analyzed using DNA binding dyes such as propidium iodide (PI) followed by fixation of cells.^37^ Since PI also stains RNA, PI-based protocols also include the process of treating cells with RNase.^36^ Since G2- and M-phases have identical DNA contents, they could not be distinguished by differences in the DNA content. To calculate the percentages of cells in each phase of the cell cycle, various software containing mathematical models that fit the DNA histogram of a singlet have been developed.^38−41^ These complex algorithms are used in modern analysis software such as FlowJo (FlowJo LLC, a subsidiary of Becton Dickinson). Besides this, doublets are excluded by plotting the height or width against the area for forward scatter or side scatter. This plot distinguished doublets (containing a 4n amount of DNA) from single cells (containing a 4n amount of DNA). The area and height values of cells containing 4n amounts of DNA are doubled, while the width is roughly the same as cells containing 2n amounts of DNA. Cells were seeded in six-well plates. Cells were synchronized and treated with CS-5Fu-QCT NPs followed by their incubation for 12, 24, and 36 h. Then, the medium was discarded. After trypsinization, cells were collected in a 15 mL tube. Cells are fixed by dropwise addition of ethanol (70%) at 4 °C. Cells were washed in PBS (1×) and resuspended in a staining buffer (containing 35 μL of RNAase in 10 mL of PBS). Cells were then stained using PI and analyzed by flow cytometry (BD Bioscience).

##### Western Blot Analysis of Expression of Different Proteins

Cells were seeded and subjected to CS-5Fu-QCT NPs. Cells were washed three times with 1× PBS before being lysed in a protease inhibitor containing RIPA buffer. The protein concentration was determined using the Micro BCA Protein Assay kit (Thermo Scientific). SDS-PAGE was used to fractionate cell lysates, which were then electrophoretically transferred to a PVDF membrane and conjugated with specific primary antibodies of caspase 3 (Cat. No. sc-56053, SCBT), BAX (Cat. No. sc-7480, SCBT), Bcl2 (Cat. No. sc-7382, SCBT), cyclin D1 (Cat. No. sc-8396, SCBT), Cdk4 (Cat. No. sc-166373, SCBT), p21 (Cat. No. sc-6246, SCBT), and p53 (Cat. No. sc-47698, SCBT). The membranes were then incubated with HRP-conjugated secondary antibodies, followed by ChemiDoc detection of chemiluminescence (ECL kit; BioRad) (Azure Biosystems).

##### Statistical Analysis

All values are represented as mean ± SEM (*n* = 3). Statistical significance was calculated using three-way or two-way ANOVA followed by post hoc analysis between the control and treatment groups. All tests were performed using Prism version 9 (GraphPad Software, San Diego, CA). When P values were found to be <0.05, the results were considered statistically significant.

## Results and Discussion

### Characterization of CS-5Fu-QCT NPs

**Figure 1:**
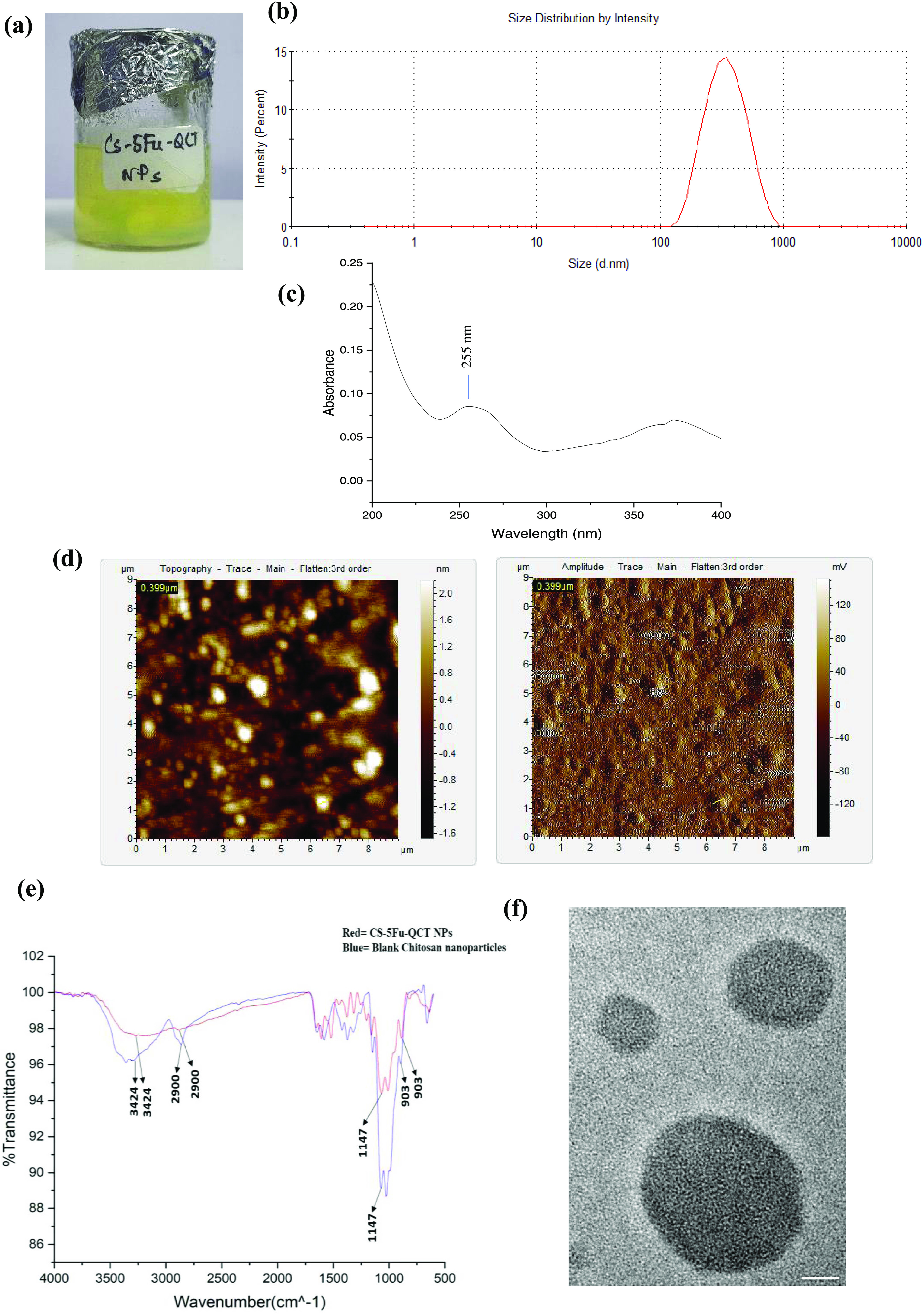
Biophysical characteristics of quercetin and 5-Fu-loaded chitosan nanoparticles (CS-5-Fu-QCT NPs). (a) Nanoparticles were prepared by the ionic gelation method. (b) Dynamic light scattering (DLS) studies of CS-5-Fu-QCT nanoparticles-size distribution by intensity. (c) UV–visible spectroscopy of CS-5-Fu-QCT NPs. (d) Determination of surface topology of CS-5-Fu-QCT NPs using atomic force microscopy (AFM). (e) FTIR analysis of CS-5-Fu-QCT NPs and blank chitosan nanoparticles. (f) Transmission microscopic image (50 nm scale bar) of CS-5-Fu-QCT NPs.

Colon cancer is one of the most common malignancies of the gastrointestinal tract and the second highest leading cause of death worldwide.^42^ Advances in nanomedicine and proper designing of nanoparticles promoted improved drug delivery and rational drug designing.^43^ Recent studies revealed that 5-Fu is the most widely used antimetabolite chemotherapeutic drug. Therefore, a suitable drug delivery system for 5-Fu can be used to achieve better therapeutic efficacy.^44^ The anticancer effect of QCT is well-recognized, and a nanoparticle-based approach enhances its anticancer activity.^45^ In order to deliver the drug effectively in a sustained release manner, chemotherapeutic drugs 5-Fu and QCT were incorporated in chitosan using the ionic gelation technique. In acidic pH, the amino group of chitosan undergoes protonation and is cross-linked with the phosphate group of negatively charged STTP. CS-5Fu-QCT nanoparticles exhibited a yellowish color (Figure 1a). Data from dynamic light scattering (DLS) revealed that the prepared CS-5Fu-QCT NPs exhibited a size of about 362.3 ± 135.5 nm (Figure 1b) with a polydispersity index of 0.189, suggesting a low polydispersity. Owing to the small size of nanoparticles, they can accumulate in the tumor site. CS-5Fu-QCT NPs were subjected to UV–visible spectrophotometric analysis, and the results displayed an absorbance peak at 255 nm corresponding to the peak of chitosan, suggesting encapsulation of two drugs in chitosan, forming CS-5FU-QCT NPs^46^ (Figure 1c). The atomic force microscopy (AFM) reveals the surface morphology of the nanoparticles. The results showed that the prepared CS-5Fu-QCT NPs had a fairly smooth surface with a spherical shape and an average particle size ranging from 350 to 400 nm (Figure 1d).

**Figure 2:**
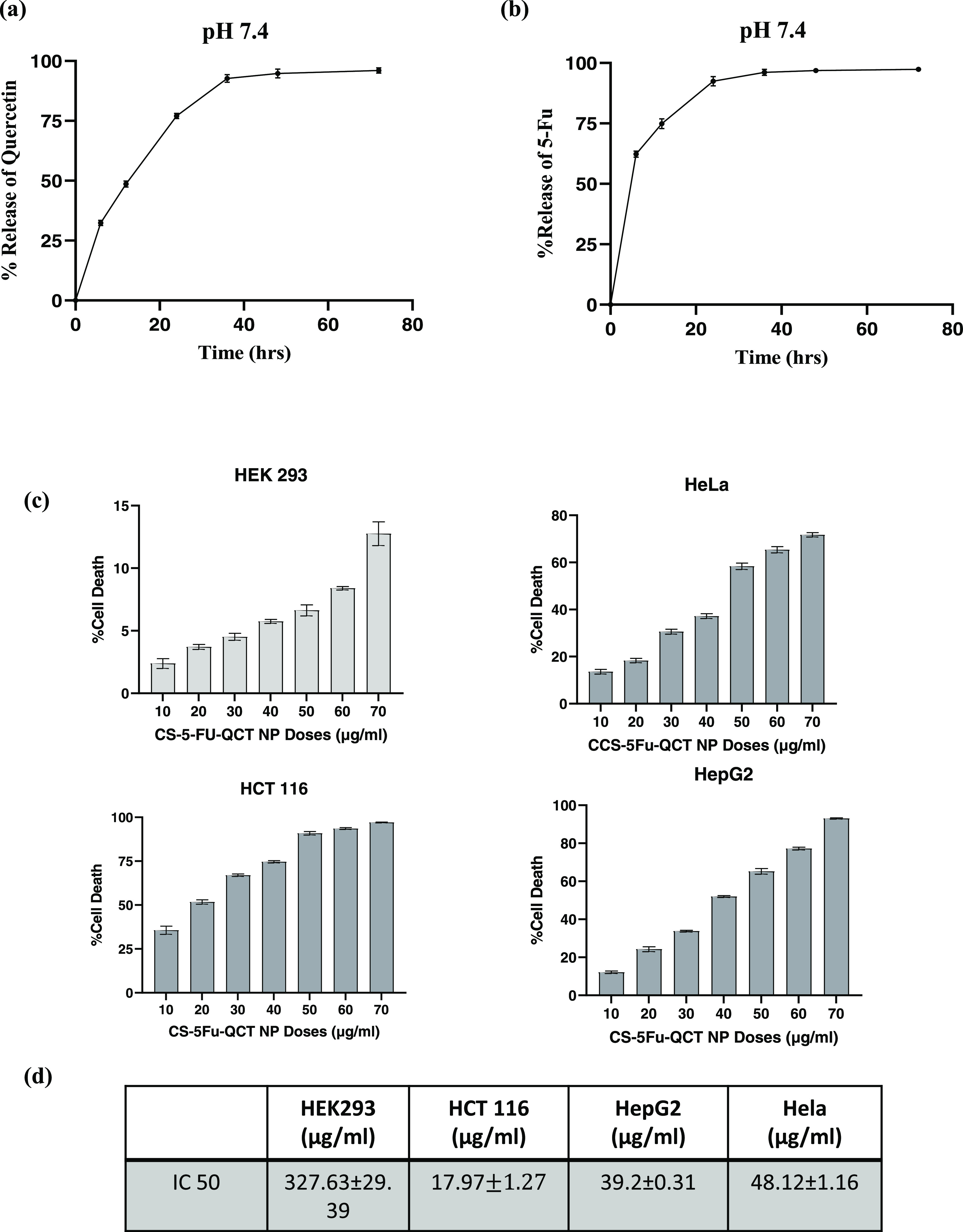
Drug release kinetics and cytotoxicity of the prepared CS-5-Fu-QCT NPs. (a) Percentage of release of quercetin from CS-5-Fu-QCT NPs. (b) Percentage of release of 5-Fu from CS-5-Fu-QCT NPs. (c) Analysis of cytotoxicity of CS-5-Fu-QCT NPs using the MTT assay. (d) IC50 of CS-5-Fu-QCT NPs on different cancer cells and human embryonic kidney cells. Data are represented as the mean ± SEM (*n* = 3).

FTIR analysis was performed for both pure CS and CS-5Fu-QCT nanoparticles to analyze the various functional groups. The FTIR spectra showed a broad peak at 3424 cm^–1^ (O–H stretching) and 2900 cm^–1^ (C–H stretching) in the spectra of chitosan (Vignesh et al., 2018). The FTIR spectra of CS-5Fu-QCT nanoparticles showed peaks at 3424 cm^–1^ (O–H stretching), 2900 cm^–1^ (C–H stretching), 903 cm^–1^ (due to aromatic −CH stretching of quercetin), and 1147 cm^–1^ (due to C–F stretching of 5-Fu). These peaks showed the presence of both 5-Fu and QCT in chitosan nanoparticles (Figure 1e).^47^ Transmission microscopic analysis of the prepared nanoparticles showed a spherical morphology of the nanoparticles with an average particle size ranging from 350 to 400 nm (Figure 1f). To maximize the encapsulation efficiency, first QCT and 5-Fu were subjected to different ratios of Cs and their maximum encapsulation was further used to optimize the encapsulation of both the drugs into Cs. Maximum encapsulation efficiencies of 94.38 ± 1.92 and 72.09 ± 2.86 were obtained for 5-Fu and QCT, respectively, when loaded together into nanocarriers. The individual loadings for 5-fluorouracil and quercetin were calculated to be 9.55 ± 0.01 and 4.62 ± 0.02, respectively. In order to analyze the nature of release of two compounds (QCT and 5-Fu), a drug release study was carried out in buffer with pH 7.4. The cumulative drug release profile of 5-Fu-QCT-loaded Cs nanoparticles exhibited the release of more than 90% QCT and 5-Fu within 36 and 24 h, respectively, followed by a slower persistent release. 5-Fu showed a much faster release than QCT within the first 6 h of the study; however, this was followed by a slower release of both the compounds forming a plateau (Figure 2a&b). This indicates the sustained release of compounds from the nanocarrier.

### CS-5Fu-QCT Nanoparticles Exerted Cytotoxic Effect on Different Cancer Cells

In order to assess the cytotoxicity of CS-5Fu-QCT nanoparticles, an MTT assay was performed in a dose-dependent manner in three different human cancer cell lines, i.e., HepG2, HCT116, and HeLa, and also the human embryonic kidney cell line Hek293 following treatment with CS-5Fu-QCT nanoparticles. Untreated cells were considered the control group. IC_50_ values for different cells were calculated and are represented in a table (Figure 2d). Results indicated a higher cytotoxic potential of CS-5Fu-QCT nanoparticles in HCT116 cells than HepG2 and HeLa cells. The IC50 value for HCT116 was 17.97 ± 1.27 μg/mL, which was much lower than those of HepG2 (IC_50_: 39.2 ± 0.31 μg/mL) and HeLa (IC_50_: 48.12 ± 1.16 μg/mL). However, no significant cytotoxicity was observed in the normal human embryonic kidney cell line since it showed less than 15% cell mortality at the highest administered dose (i.e., 70 μg/mL) of CS-5Fu-QCT NPs for all types of cells used in this experiment. As a result, the dose–response graph was extrapolated to obtain the approximate IC50 value of CS-5Fu-QCT NPs for HEK293 cells. The IC_50_ value of HEK293 was calculated to be 327.63 ± 29.39 μg/mL. Hence, the HCT116 cell line was used for further studies and a 17.97 ± 1.27 μg/mL dose of CS-5Fu-QCT nanoparticles was used for future experiments.

### CS-5Fu-QCT Nanoparticles Induced Apoptosis *In Vitro* in HCT116 Cells

**Figure 3:**
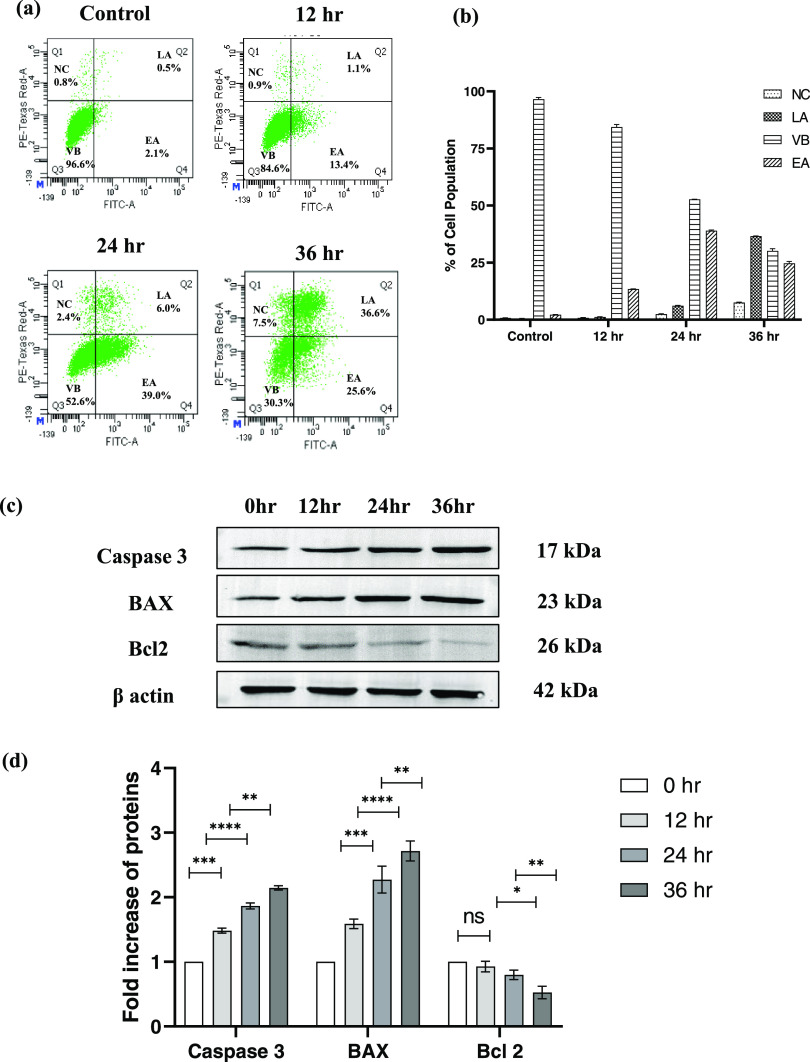
Induction of apoptosis in HCT116 cells by CS-5-Fu-QCT NPs. (a) Analysis of apoptosis by Annexin V/FITC followed by time-dependent administration of CS-5-Fu-QCT NPs. (b) Representative bar diagram indicating apoptosis at different time intervals. (c) Western blot of proapoptotic protein BAX and caspase 3 and antiapoptotic Bcl2. (d) Densitometric analysis of the expression of pro- and antiapoptotic proteins. Data are represented as the mean ± SEM (*n* = 3).

In order to find the apoptotic potential of the prepared NPs, and to find whether its action is time-dependent, HCT116 cells were treated with the prepared NPs and incubated for different time intervals, followed by their analysis using an Annexin V/FITC apoptosis detection kit using flow cytometry, and the results revealed that cells exposed to CS-5Fu-QCT nanoparticles have shown significant elevation in necrotic (7.5%), early apoptotic (25.6%), and late apoptotic (36.6%) populations after 36 h exposure compared to 12 h (necrotic—0.9%, early apoptotic—13.4%, and late apoptotic—1.1%) and 24 h exposure (necrotic—2.4%, early apoptotic—39.0%, and late apoptotic—6.0%). However, the control cells did not show significant apoptosis or necrosis. Increases in the percentages of necrotic, early apoptotic, and late apoptotic populations upon administration of CS-5Fu-QCT NPs supports the apoptotic induction potential of CS-5Fu-QCT NPs in a time-dependent manner (Figure 3a&b).

**Figure 4:**
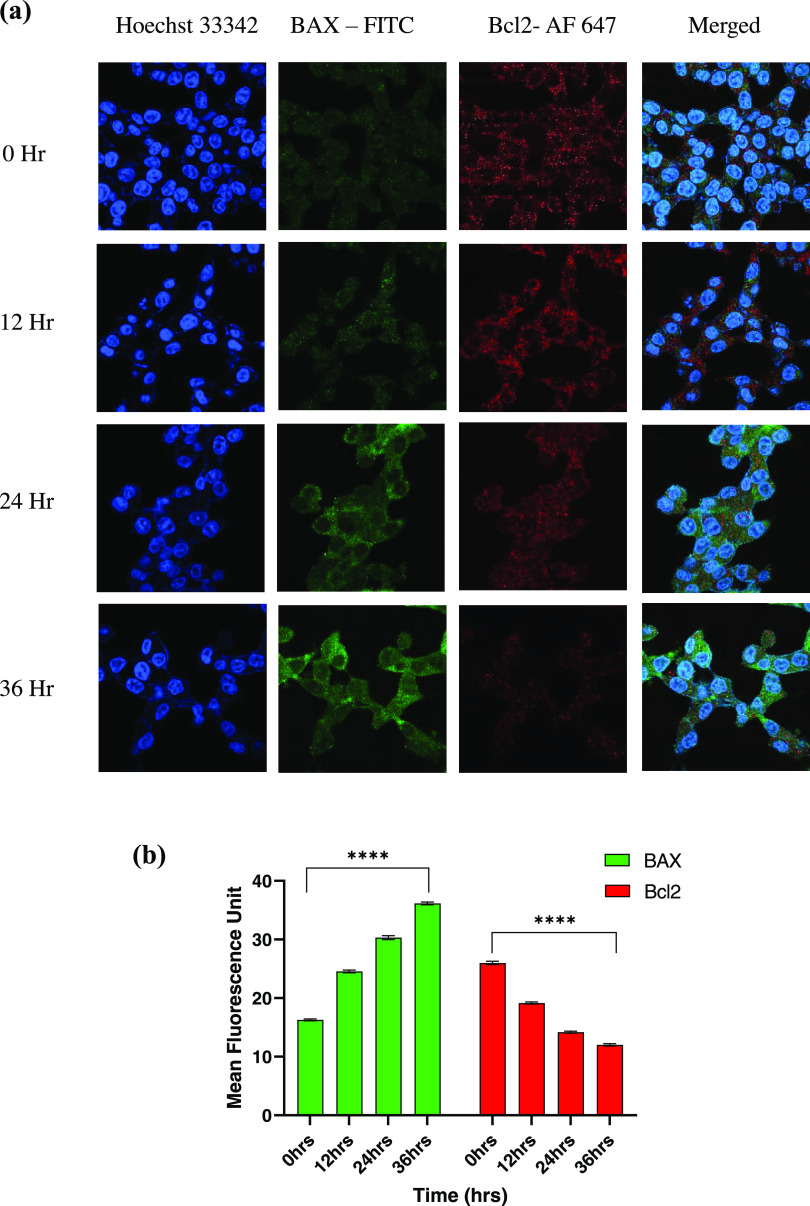
CS-5-Fu-QCT NPs altered the expression of proteins associated with apoptosis. (a) Confocal microscopic analysis of the proapoptotic protein BAX and the antiapoptotic protein Bcl2 in HCT116 cells followed by administration of CS-5-Fu-QCT NPs in a time-dependent manner. (b) Bar graph showing the comparative expression of BAX and Bcl2 proteins.

BAX and caspase 3 are proapoptotic proteins directly involved in the progression of apoptosis, while Bcl2 imparts an opposite action resulting in blockage of apoptosis. Western blot analysis of these key proteins associated with cellular apoptosis indicated a time-dependent increase of caspase 3 and BAX proteins, indicative of an increase in apoptosis (12 h < 24 h1 < 36 h) and a decrease in expression of Bcl2 (12 h > 24 h > 36 h) following CS-5Fu-QCT NP administration, suggesting induction of apoptosis by CS-5Fu-QCT NPs (Figure 3c&d). Confocal microscopic analysis of BAX and Bcl2 also supports the finding of Western blot analysis (Figure 4a&b).

### CS-5Fu-QCT Nanoparticles Were Involved in Endogenous ROS Generation and Affected Cell Proliferation

**Figure 5:**
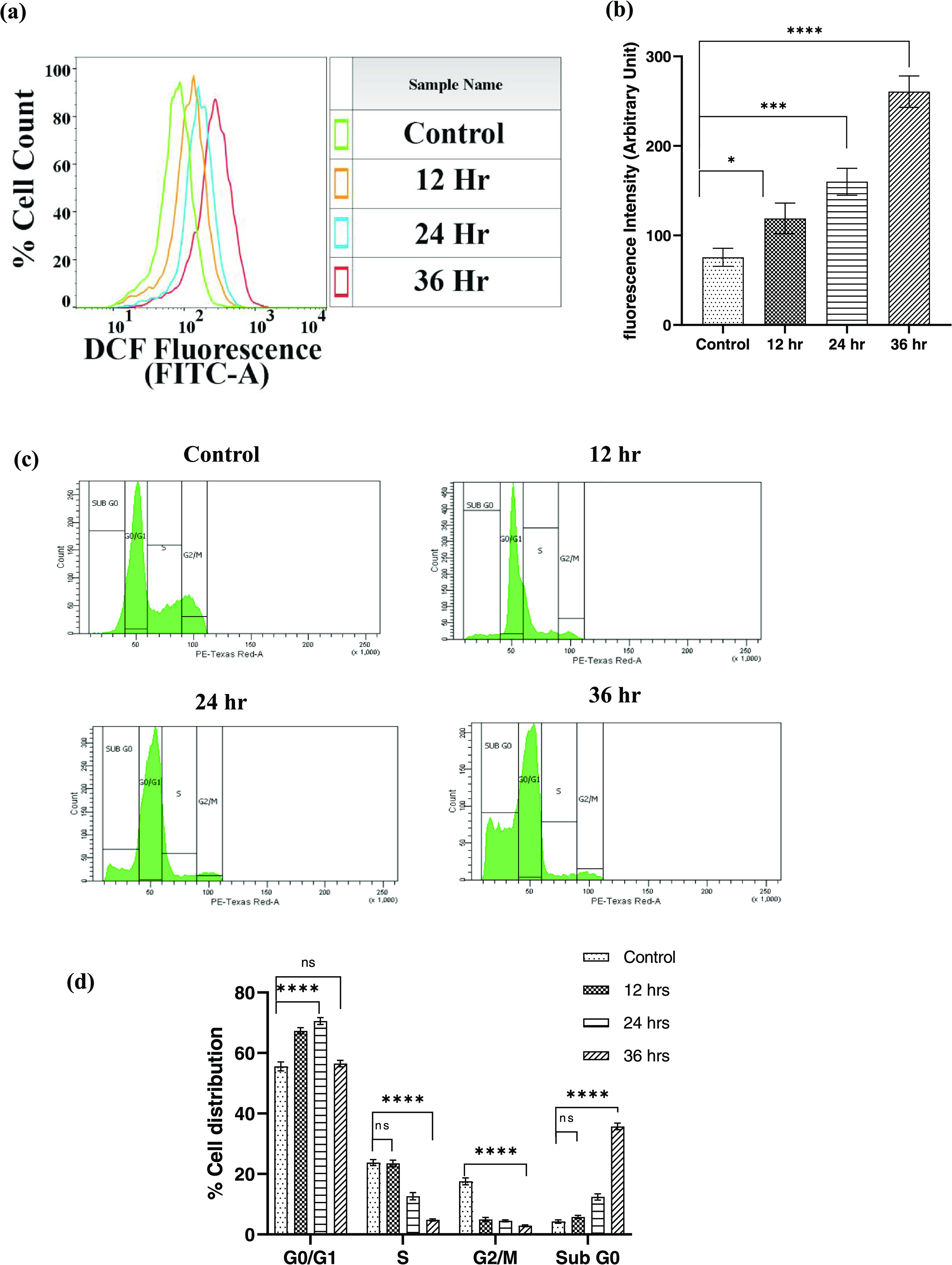
CS-5-Fu-QCT NPs caused ROS generation and arrested cell cycle in HCT116 cells. (a) Analysis of intracellular ROS using DCFDA fluorescence via flow cytometry. (b) Representative bar diagram of intracellular ROS generation. (c) Analysis of cell cycle using PI followed by treatment with CS-5-Fu-QCT NPs. (d) Representative bar diagram of percent distribution of cells across different phases of the cell cycle. Data are represented as the mean ± SEM (*n* = 3).

A variety of extrinsic and intrinsic signals including ROS, DNA-damaging agents, and viral infections can trigger apoptosis.^48^ Abnormal generation of ROS results in alteration of cellular functions and leads to DNA damage, cell-cycle arrest, lipid peroxidation, activation of caspase cascade, and eventually apoptosis.^49^ Different studies have illuminated nanoparticle-induced ROS generation as an important mediator to cellular damage and apoptosis. Hence, the level of intracellular ROS was determined using flow cytometry following administration of CS-5FU-QCT NPs *in vitro and* DCFDA fluorescence was used to track the intracellular oxidative environment at different time intervals following administration of CS-5Fu-QCT NPs. H_2_DCF-DA enters the viable cells via the plasma membrane and oxidizes to its fluorescent form. Results showed a dose-dependent increase in DCFDA fluorescence, indicating an increase in the level of intracellular ROS. Cells treated with CS-5Fu-QCT NPs had shown 1.14, 1.44, and 1.73-fold increases in cellular ROS on 12, 24, and 36 h after administration of CS-5Fu-QCT NPs, respectively, compared to the control (Figure 5a&b).

The cell cycle in eukaryotic cells consists of four phases. The G1 phase induces the cells to undergo proliferation by protein synthesis followed by their transition to the S phase when DNA replication takes place. After these events, when the cells enter the G2 phase, they prepare for mitosis (M). The precise transition from G1 to S phases of the cell cycle is critical for controlling eukaryotic cell proliferation, and its disruption promotes oncogenesis.^50^ Anticancer drugs that target the cell cycle cause cell death by blocking the cell cycle at specific checkpoints (G0/G1 phase, S phase, and G2/M phase, sub-G0 Phase).^51^ These drugs may inhibit cell proliferation (G0/G1 arrest), DNA replication (S phase arrest), apoptosis (sub-G0 arrest), or mitosis (G2/M phase arrest).^52^ To determine the potential of CS-5Fu-QCT NPs to alter cell proliferation, the cell populations at different phases of the cell cycle were subjected to flow cytometric analysis. The selection criteria of the cells in a particular phase of the cell cycle were solely based on the relative DNA content (i.e., relative fluorescence by PI conjugation with DNA) of those cells, and the amplitude of the histogram corresponds to the number of cells at the respective phases of the cell cycle. As the data reveal, control HCT 116 cells had a higher G0/G1 population, which was followed by G2/M and S phase populations. However, treatment with CS-5Fu-QCT NPs significantly reduced G2/M and S phase populations and a concomitant increase in Sub-G0 and G0/G1 populations with increasing time. This suggests a possible hindrance in cell-cycle progression at the G0/G1 phase. According to Nunez et al., the G0/G1 block corresponds to inhibition of cell proliferation and the increase in the Sub-G0 population corresponds to an increase in the apoptotic population.^53^ Therefore, it can be interpreted that CS-5Fu-QCT NPs had the potential to cease the cell cycle at the G0/G1 phase followed by apoptosis of HCT 116 cells (Figure 5d).

### CS-5Fu-QCT Nanoparticles Altered the Expression of Major Proteins Associated with Cell Cycle

**Figure 6:**
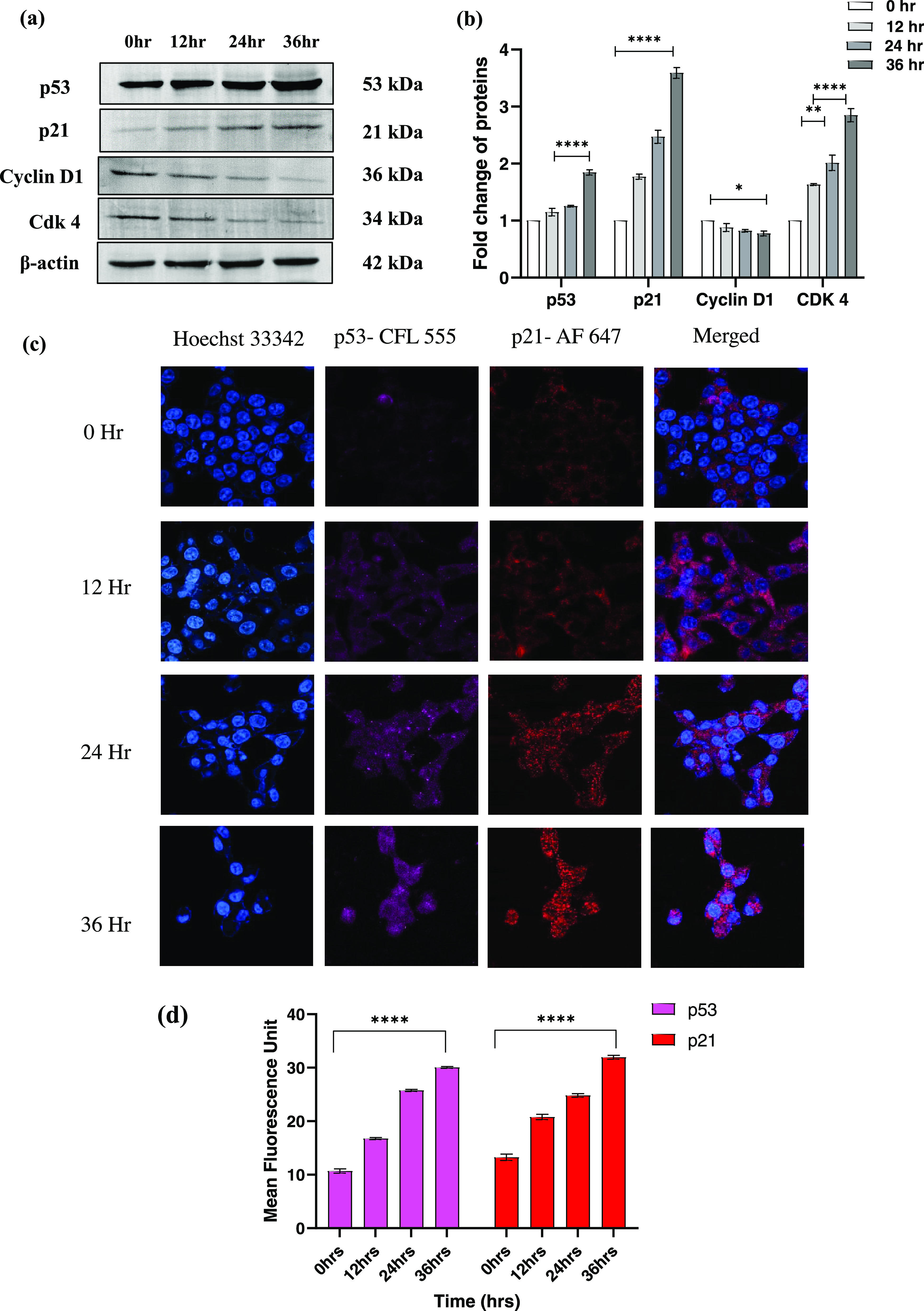
CS-5-Fu-QCT NPs modulated the expression of proteins associated with cell-cycle regulation. (a) Western blot analysis of p53, p21, cyclin D, and Cdk4. (b) Representative bar diagram of expression of proteins in Western blot analysis. (c) Confocal microscopic analysis of p53 and p21. Data are represented as the mean ± SEM (*n* = 3). (d) Bar diagram showing the comparative expression of P53 and P21.

In order to confirm the arrested phase of the cell cycle, Western blot analysis was performed to assess the expression of key regulatory proteins associated with the G0/G1 phase of the cell cycle, i.e., p53, p21, Cyclin D, and Cdk4. Results displayed upregulation of p53 and p21 and downregulation of Cyclin D and Cdk4, which corresponds to the flow cytometric analysis of G0/G1 phase arrest (Figure 6a&b). A similar pattern of expression is noted for p53 and p21 by confocal microscopic analysis, indicating CS-5FU-QCT NPs mediated G0/G1 arrest (Figure 6c&d).

## Conclusions

In summary, CS-5FU-QCT NPs showed a promising approach towards chemotherapeutics by effectively inducing cytotoxicity and apoptosis of the HCT116 cells through endogenous ROS generation. Moreover, it also modulated the p53/p21 axis, causing the G0/G1 phase arrest of the cell cycle, thereby inducing apoptosis via alteration of key proteins of the apoptotic pathway.

## Supporting Information Available

Figure S1, molecular arrangements of CS-5Fu-QCT NPs; Figure S2, whole blot data of Western blot (of Figure 3c in the main manuscript); Figure S3, full blots of Western blot data (Figure 6a in the main manuscript); Scheme S1, gating strategy for flow cytometry (PDF)

## References

[ref1] Khan I.; Saeed K.; Khan I. Nanoparticles: Properties, applications and toxicities. Arabian J. Chem. 2019, 12 (7), 908–931. 10.1016/j.arabjc.2017.05.011.

[ref2] Modak A.; Barui A. K.; Ranjan Patra C.; Bhaumik A. A luminescent nanoporous hybrid material based drug delivery system showing excellent theranostics potential for cancer. Chem. Commun. 2013, 49 (69), 7644 10.1039/c3cc43487g. 23872783

[ref3] Patra J. K.; Das G.; Fraceto L. F.; et al. Nano based drug delivery systems: Recent developments and future prospects 10 Technology 1007 Nanotechnology 03 Chemical Sciences 0306 Physical Chemistry (incl. Structural) 03 Chemical Sciences 0303 Macromolecular and Materials Chemistry 11 Medical and Health Sciences 1115 Pharmacology and Pharmaceutical Sciences 09 Engineering 0903 Biomedical Engineering Prof Ueli Aebi, Prof Peter Gehr. J. Nanobiotechnology. 2018, 16 (1), 71 10.1186/s12951-018-0392-8. 30231877 PMC6145203

[ref4] Zhang N.; Yin Y.; Xu S. J.; Chen W. S. 5-Fluorouracil: Mechanisms of resistance and reversal strategies. Molecules 2008, 13 (8), 1551–1569. 10.3390/molecules13081551. 18794772 PMC6244944

[ref5] WHO. Cancer. https://www.who.int/news-room/fact-sheets/detail/cancer.

[ref6] Siegel R. L.; Miller K. D.; Wagle N. S.; Jemal A. Cancer statistics, 2023. CA Cancer J. Clin. 2023, 73 (1), 17–48. 10.3322/caac.21763. 36633525

[ref7] Tian H.; Zhang T.; Qin S.; et al. Enhancing the therapeutic efficacy of nanoparticles for cancer treatment using versatile targeted strategies. J. Hematol. Oncol. 2022, 15 (1), 132 10.1186/s13045-022-01320-5. 36096856 PMC9469622

[ref8] Mohammed M.; Syeda J.; Wasan K.; Wasan E. An Overview of Chitosan Nanoparticles and Its Application in Non-Parenteral Drug Delivery. Pharmaceutics. 2017, 9 (4), 53 10.3390/pharmaceutics9040053. 29156634 PMC5750659

[ref9] Valencia-Lazcano A. A.; Hassan D.; Pourmadadi M.; et al. 5-Fluorouracil nano-delivery systems as a cutting-edge for cancer therapy. Eur. J. Med. Chem. 2023, 246, 114995 10.1016/j.ejmech.2022.114995. 36493619

[ref10] Hassan Y. A.; Alfaifi M. Y.; Shati A. A.; Elbehairi S. E. I.; Elshaarawy R. F. M.; Kamal I. Co-delivery of anticancer drugs via poly(ionic crosslinked chitosan-palladium) nanocapsules: Targeting more effective and sustainable cancer therapy. J. Drug Delivery Sci. Technol. 2022, 69, 103151 10.1016/j.jddst.2022.103151.

[ref11] Canivenc-Lavier M. C.; Vernevauta M. F.; Totisb M.; Siess’ M. H.; Magdaloub J.; Suscheteta M. Comparative Effects of Flavonoids and Model Inducers on Drug-Metabolizing Enzymes in Rat Liver. Toxicology 1996, 114, 19–27. 10.1016/s0300-483x(96)03412-9. 8931757

[ref12] Shih H.; Pickwell G. V.; Quattrochi L. C. Differential Effects of Flavonoid Compounds on Tumor Promoter-Induced Activation of the Human CYP1A2 Enhancer. Arch. Biochem. Biophys. 2000, 373, 287–294. 10.1006/abbi.1999.1550. 10620351

[ref13] Moon Y. J.; Wang X.; Morris M. E. Dietary flavonoids: Effects on xenobiotic and carcinogen metabolism. Toxicol. In Vitro 2006, 20 (2), 187–210. 10.1016/j.tiv.2005.06.048. 16289744

[ref14] Jeong J. H.; An J. Y.; Kwon Y. T.; Rhee J. G.; Lee Y. J. Effects of low dose quercetin: Cancer cell-specific inhibition of cell cycle progression. J. Cell Biochem. 2009, 106 (1), 73–82. 10.1002/jcb.21977. 19009557 PMC2736626

[ref15] Formica J. V.; Regelsont W. Review of the Biology of Quercetin and Related Bioflavonoids. Food Chem. Toxicol. 1995, 33, 1061–1080. 10.1016/0278-6915(95)00077-1. 8847003

[ref16] Galluzzo P.; Martini C.; Bulzomi P.; et al. Quercetin-induced apoptotic cascade in cancer cells: Antioxidant versus estrogen receptor α-dependent mechanisms. Mol. Nutr. Food Res. 2009, 53 (6), 699–708. 10.1002/mnfr.200800239. 19194971

[ref17] Davatgaran-Taghipour Y.; Masoomzadeh S.; Farzaei M. H.; et al. Polyphenol nanoformulations for cancer therapy: Experimental evidence and clinical perspective. Int. J. Nanomed. 2017, 12, 2689–2702. 10.2147/IJN.S131973. PMC5388197 28435252

[ref18] Roy S.; Rhim J. W. Fabrication of chitosan-based functional nanocomposite films: Effect of quercetin-loaded chitosan nanoparticles. Food Hydrocolloids 2021, 121, 107065 10.1016/j.foodhyd.2021.107065.

[ref19] Focaccetti C.; Bruno A.; Magnani E.; et al. Effects of 5-fluorouracil on morphology, cell cycle, proliferation, apoptosis, autophagy and ros production in endothelial cells and cardiomyocytes. PLoS One 2015, 10 (2), e0115686 10.1371/journal.pone.0115686. 25671635 PMC4324934

[ref20] Fonseca-Silva F.; Inacio J. D. F.; Canto-Cavalheiro M. M.; Almeida-Amaral E. E. Reactive oxygen species production and mitochondrial dysfunction contribute to quercetin induced death in Leishmania amazonensis. PLoS One 2011, 6 (2), e14666 10.1371/journal.pone.0014666. 21346801 PMC3035610

[ref21] Schumacker P. T. Reactive oxygen species in cancer cells: Live by the sword, die by the sword. Cancer Cell 2006, 10 (3), 175–176. 10.1016/j.ccr.2006.08.015. 16959608

[ref22] Waris G.; Ahsan H. Reactive oxygen species: Role in the development of cancer and various chronic conditions. J. Carcinog. 2006, 5, 14 10.1186/1477-3163-5-14. 16689993 PMC1479806

[ref23] Buechter D. D. Free Radicals and Oxygen Toxicity. Pharm. Res. 1988, 5, 253–260. 10.1023/a:1015914418014. 3072554

[ref24] Redza-Dutordoir M.; Averill-Bates D. A. Activation of apoptosis signalling pathways by reactive oxygen species. Biochim. Biophys. Acta, Mol. Cell Res. 2016, 1863 (12), 2977–2992. 10.1016/j.bbamcr.2016.09.012. 27646922

[ref25] Elmore S. Apoptosis: A Review of Programmed Cell Death. Toxicol. Pathol. 2007, 35, 495–516. 10.1080/01926230701320337. 17562483 PMC2117903

[ref26] Srivastava S.; Somasagara R. R.; Hegde M.; et al. Quercetin, a natural flavonoid interacts with DNA, arrests cell cycle and causes tumor regression by activating mitochondrial pathway of apoptosis. Sci. Rep. 2016, 6, 24049 10.1038/srep24049. 27068577 PMC4828642

[ref27] MHAIDAT N. M.; BOUKLIHACENE M.; THORNE R. F. 5-Fluorouracil-induced apoptosis in colorectal cancer cells is caspase-9-dependent and mediated by activation of protein kinase C-δ. Oncol Lett. 2014, 8 (2), 699–704. 10.3892/ol.2014.2211. 25013487 PMC4081407

[ref28] Donjerkovic D.; Scott D. W. Regulation of the G1 Phase of the Mammalian Cell Cycle. Cell Res. 2000, 10, 1–16. 10.1038/sj.cr.7290031. 10765979

[ref29] Rather R. A.; Bhagat M. Quercetin as an innovative therapeutic tool for cancer chemoprevention: Molecular mechanisms and implications in human health. Cancer Med. 2020, 9 (24), 9181–9192. 10.1002/cam4.1411. 31568659 PMC7774748

[ref30] Gao L.; Shen L.; Yu M.; et al. Colon cancer cells treated with 5-fluorouracil exhibit changes in polylactosamine-type N-glycans. Mol. Med. Rep. 2014, 9 (5), 1697–1702. 10.3892/mmr.2014.2008. 24604396

[ref31] Choi J. H.; Yoon J. S.; Won Y. W.; Park B. B.; Lee Y. Y. Chloroquine enhances the chemotherapeutic activity of 5-fluorouracil in a colon cancer cell line via cell cycle alteration. APMIS 2012, 120 (7), 597–604. 10.1111/j.1600-0463.2012.02876.x. 22716215

[ref32] Xu J. L.; Jin B.; Ren Z. H.; et al. Chemotherapy plus Erlotinib versus Chemotherapy Alone for Treating Advanced Non-Small Cell Lung Cancer: A Meta-Analysis. PLoS One 2015, 10 (7), e0131278 10.1371/journal.pone.0131278. 26147288 PMC4493135

[ref33] Li P.; Wang Y.; Peng Z.; She F.; Kong L. Development of chitosan nanoparticles as drug delivery systems for 5-fluorouracil and leucovorin blends. Carbohydr. Polym. 2011, 85 (3), 698–704. 10.1016/j.carbpol.2011.03.045.

[ref34] Chaitra K.; Ravi Singh K.; Raghu M. S.; Sadashiva M. P.; Prashanth K. N. Mucic acid cross-linked chitosan nanoparticles as a dual drug delivery system for treatment of colorectal cancer- insilico and invitro studies. Chem. Data Collect. 2022, 41, 100928 10.1016/j.cdc.2022.100928.

[ref35] Lv Q.; Yu A.; Xi Y.; et al. Development and evaluation of penciclovir-loaded solid lipid nanoparticles for topical delivery. Int. J. Pharm. 2009, 372 (1–2), 191–198. 10.1016/j.ijpharm.2009.01.014. 19429280

[ref36] Ligasová A.; Frydrych I.; Koberna K. Basic Methods of Cell Cycle Analysis. Int. J. Mol. Sci. 2023, 24 (4), 3674 10.3390/ijms24043674. 36835083 PMC9963451

[ref37] Darzynkiewicz Z.; Juan G.; Bedner E. Determining Cell Cycle Stages by Flow Cytometry. Curr. Protoc. Cell Biol. 1999, 1 (1), Unit 8.4 10.1002/0471143030.cb0804s01. 18228389

[ref38] Nunez R. DNA Analysis Using Flow Cytometry 67 DNA Measurement and Cell Cycle Analysis by Flow Cytometry. 2001; Vol. 3. 11488413

[ref39] Dean P. N.; Jett J. H. MATHEMATICAL ANALYSIS OF DNA DISTRIBUTIONS DERIVED FROM FLOW MICROFLUOROMETRY. J. Cell Biol. 1974, 60, 523–527. 10.1083/jcb.60.2.523. 4855906 PMC2109170

[ref40] Dean P. N. A SIMPLIFIED METHOD OF DNA DISTRIBUTION ANALYSIS. Cell Proliferation 1980, 13, 299–308. 10.1111/j.1365-2184.1980.tb00468.x. 6989507

[ref41] Baisch H.; Beck Hp.; Christensen I. J.; et al. A Comparison of Mathematical Methods for the Analysis of DNA Histograms Obtained by Flow Cytometry. Cell Tissue Kinet. 1982, 15, 235–249. 10.1111/j.1365-2184.1982.tb01043.x. 7083295

[ref42] Colorectal Cancer Awareness Month 2022.

[ref43] Adir O.; Poley M.; Chen G.; et al. Integrating Artificial Intelligence and Nanotechnology for Precision Cancer Medicine. Adv. Mater. 2020, 32 (13), 1901989 10.1002/adma.201901989. PMC7124889 31286573

[ref44] Entezar-Almahdi E.; Mohammadi-Samani S.; Tayebi L.; Farjadian F. Recent advances in designing 5-fluorouracil delivery systems: A stepping stone in the safe treatment of colorectal cancer. Int. J. Nanomed. 2020, 15, 5445–5458. 10.2147/IJN.S257700. PMC7398750 32801699

[ref45] Men K.; Duan X.; Wei X.; et al. Nanoparticle-Delivered Quercetin for Cancer Therapy. Anticancer Agents Med. Chem. 2014, 14 (6), 826–832. 10.2174/1871520614666140521122932. 24851877

[ref46] Wu X.; Liu C.; Chen H.; Zhang Y.; Li L.; Tang N. Layer-by-layer deposition of hyaluronan and quercetin-loaded chitosan nanoparticles onto titanium for improving blood compatibility. Coatings 2020, 10 (3), 256 10.3390/coatings10030256.

[ref47] David K. I.; Jaidev L. R.; Sethuraman S.; Krishnan U. M. Dual drug loaded chitosan nanoparticles—sugar-coated arsenal against pancreatic cancer. Colloids Surf., B 2015, 135, 689–698. 10.1016/j.colsurfb.2015.08.038. 26340358

[ref48] Redza-Dutordoir M.; Averill-Bates D. A. Activation of apoptosis signalling pathways by reactive oxygen species. Biochim. Biophys. Acta, Mol. Cell Res. 2016, 1863 (12), 2977–2992. 10.1016/j.bbamcr.2016.09.012. 27646922

[ref49] Forman H. J.; Torres M.; Fukuto J. Redox Signaling. Mol. Cell. Biochem. 2002, 234-235, 49–62. 12162460

[ref50] Bertoli C.; Skotheim J. M.; De Bruin R. A. M. Control of cell cycle transcription during G1 and S phases. Nat. Rev. Mol. Cell Biol. 2013, 14 (8), 518–528. 10.1038/nrm3629. 23877564 PMC4569015

[ref51] Stewart Z. A.; Westfall M. D.; Pietenpol J. A. Cell-cycle dysregulation and anticancer therapy. Trends Pharmacol. Sci. 2003, 24 (3), 139–145. 10.1016/S0165-6147(03)00026-9. 12628359

[ref52] Nunez R. DNA Analysis Using Flow Cytometry 67 DNA Measurement and Cell Cycle Analysis by Flow Cytometry. 2001; Vol. 3. 11488413

[ref53] Nunez R. DNA Measurement and Cell Cycle Analysis by Flow Cytometry. Curr. Issues Mol. Biol. 2001, 3, 67–70. 10.21775/cimb.003.067. 11488413

