# Supplementary material for: Quercetin and 5-Fu Loaded Chitosan Nanoparticles Trigger Cell-Cycle Arrest and Induce Apoptosis in HCT116 Cells via Modulation of the p53/p21 Axis
Source: ACS Omega. 2023 Sep 28;8(40):36893–905. doi: 10.1021/acsomega.3c03933 (PMC10569019; doi:10.1021/acsomega.3c03933)

Quercetin and 5-FU loaded chitosan nanoparticles triggers cell cycle arrest and induce apoptosis in HCT116 Cells via modulation of p53/p21 axis

Authors: Sanjib Das [1#], Moumita Saha [1#], Lokesh Chandra Mahata[2], Arya China[2], Niloy Chatterjee[3,4], Krishna Das Saha [1,*]

Contact Information:

- Sanjib Das [1] (sanjibsanjib7@gmail.com)
- Moumita Saha [1] (moumitasahaiicb@gmail.com)
- Lokesh Chandra Mahata [2] (mahatalokesh99@gmail.com)
- Arya China [2] (arya1998china@gmail.com)
- Niloy Chatterjee [3,4] (chatterjeenil99@gmail.com)

***Corresponding Author:**

- Krishna Das Saha [1,*] (Krishna@iicb.res.in)

# Denotes equal contribution

[1] Cancer Biology and Inflammatory Disorder division, CSIR- Indian Institute of Chemical Biology, Jadavpur, Kolkata, West Bengal, India, 700032

[2] Department of Pharmaceutical Technology, Maulana Abul Kalam Azad University of Technology, Haringhata, Nadia, West Bengal, India, 741249

[3] Laboratory of food Science and Technology, Food and Nutrition, University of Calcutta, 20B, Judges Court Road, Kolkata, West Bengal, India, 700027

[4] Centre for Research in Nanoscience & Nanotechnology, University of Calcutta, JD-2, Sector-III, Salt Lake City, Kolkata, West Bengal, India, 700098

Supplementary Information:

Figure S1: Chemical Composition and molecular arrangements of CS-5Fu-QCT NPs. Chitosan produces crosslinks in presence of sodium tri polyphosphate, Quercetin and 5-FU gets encapsulated within the crosslinks.

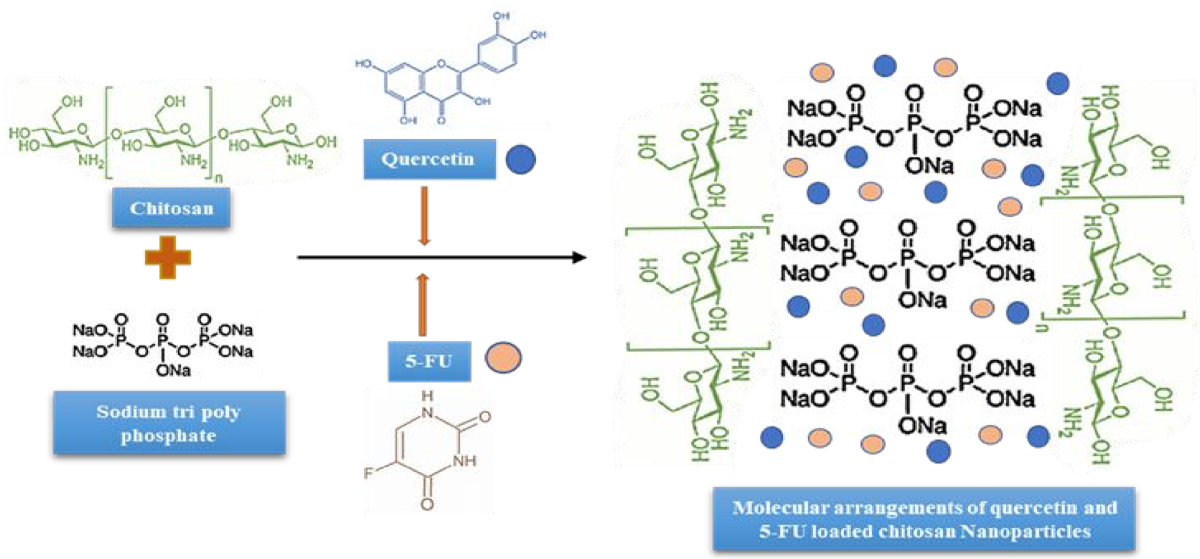

Figure S2: Full Blots of western Blot data (Figure 3c in main manuscript). Western Blot analysis of pro-apoptotic protein BAX and Caspase 3 and anti-apoptotic protein Bcl2.

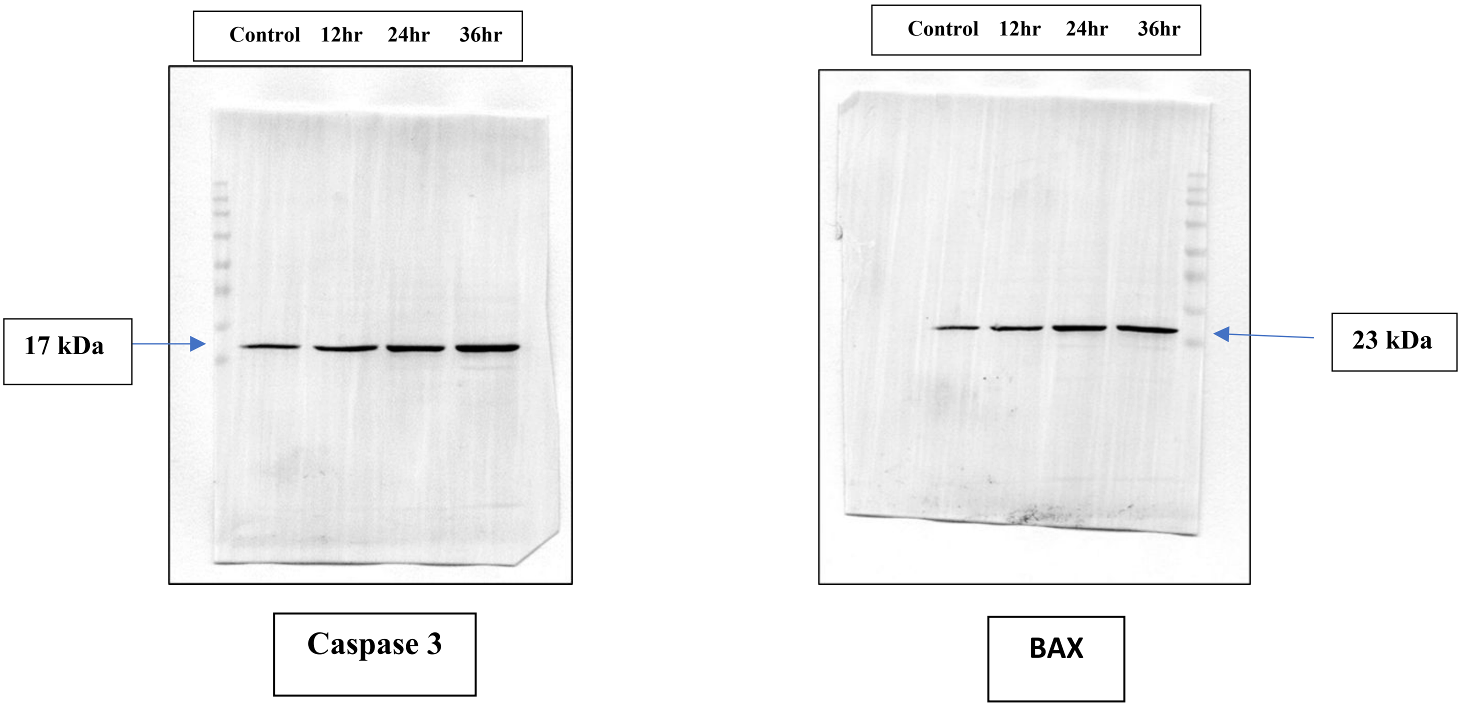

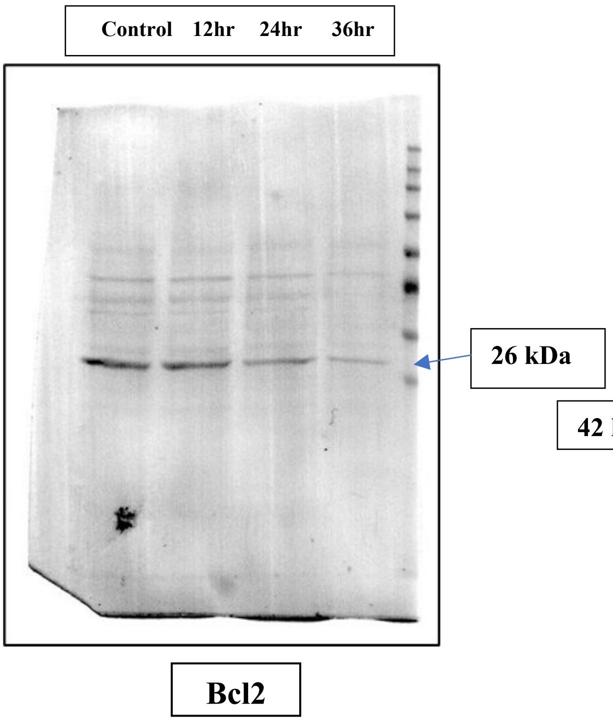

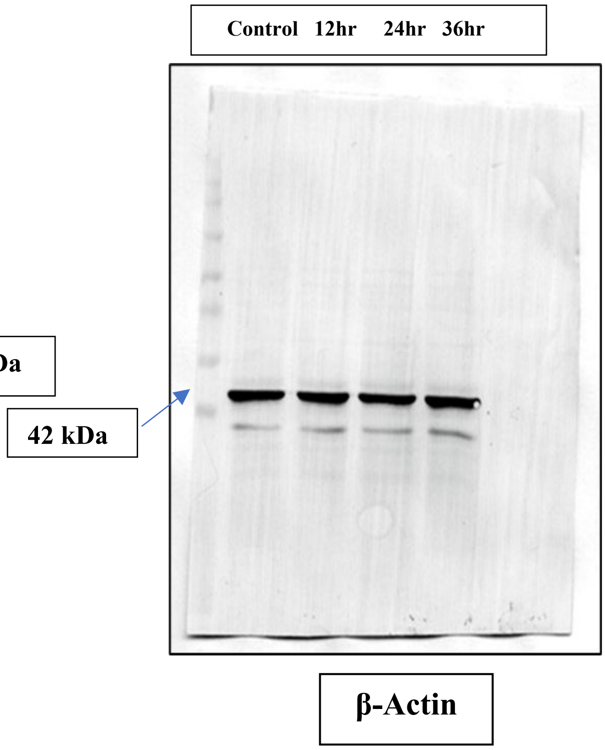

Figure S3: Full Blots of western Blot data (Figure 6a in main manuscript). Western Blot analysis of key regulatory proteins of cell cycle i.e., p53, p21, Cyclin D and Cdk4. P53 and p21 modulates the fate of cell cycle while, Cyclin D and Cdk4 is the marker for G1 phase of cell cycle.

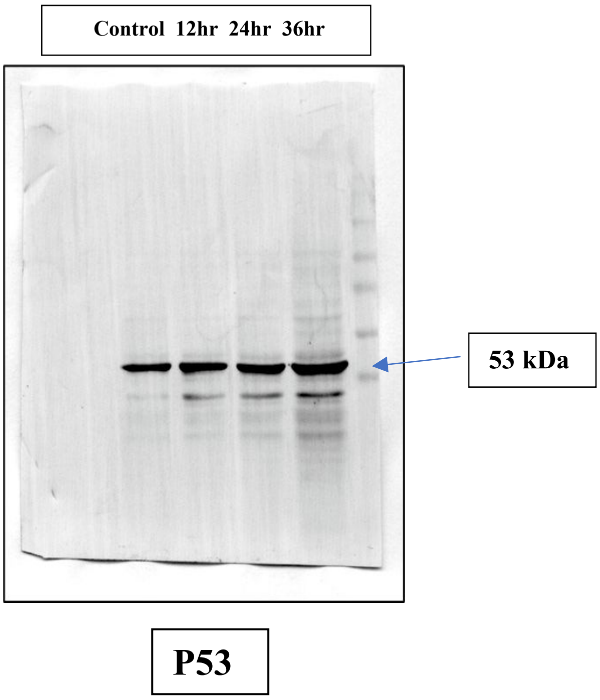

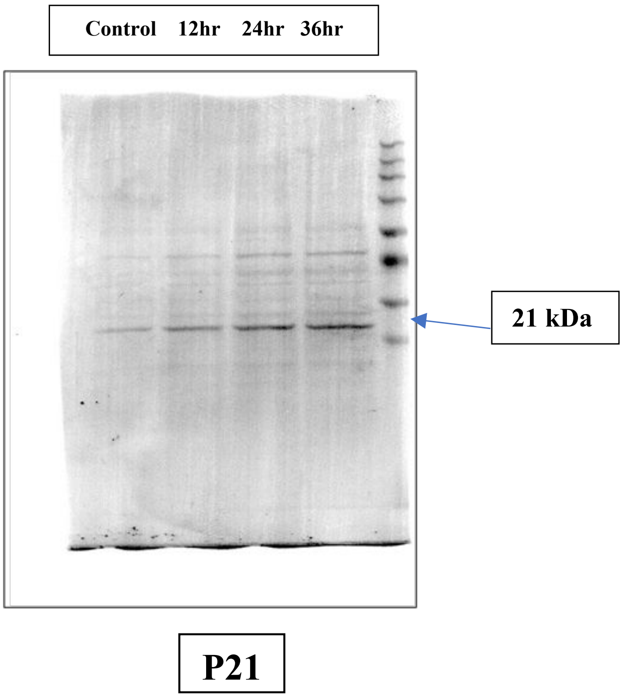

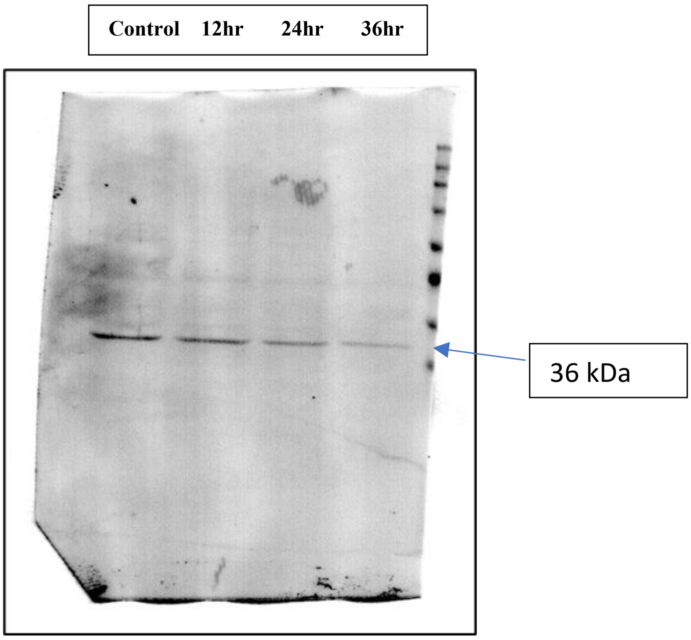

Cyclin D1

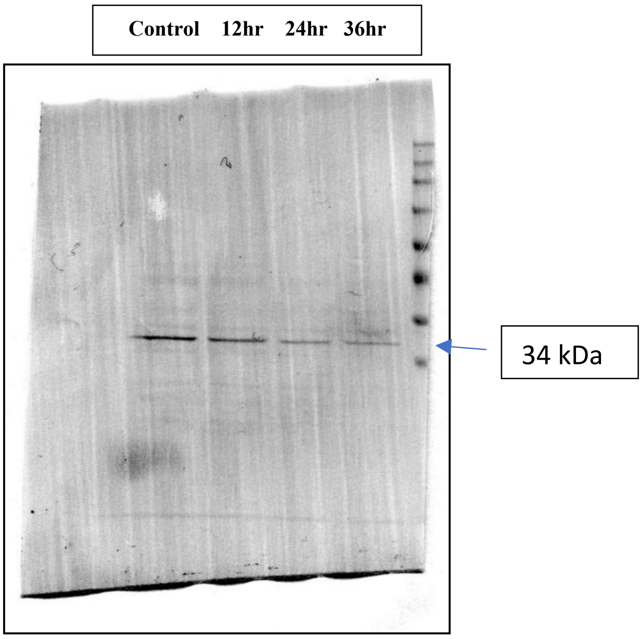

Cdk4

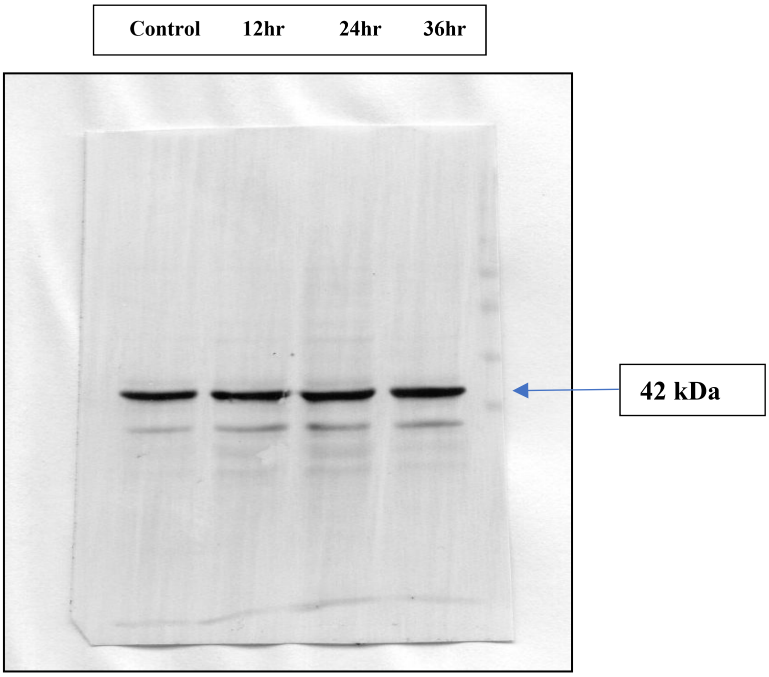

β- actin

Scheme S1: Gating strategy employed in flowcytometry:

All of our flowcytometric experiments were performed based on the selection of cells followed by doublet exclusion. To identify cells of interest based on size and granularity (complexity), forward versus side scatter (FSC vs SSC) gating was commonly used. It is commonly assumed that forward scatter indicates cell size, whereas side scatter indicates cell complexity or granularity. This gating was followed by selecting the singlets by plotting the height or width against the area for forward scatter or side scatter. Cells were then analysed by modern analysis software for flowcytometry which are integrated with complex mathematical algorithms to obtain the percentage of desired cell population. An example of the same is given in the following figure.

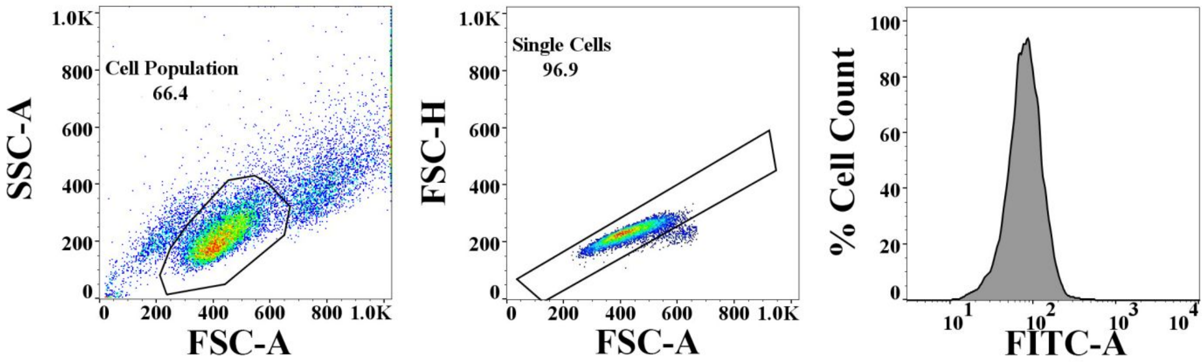

Supplement: Supplementary file 1 — ao3c03933_si_001.pdf [file ao3c03933_si_001.pdf]
